# The mediating role of perceived organizational support in the association between challenge-hindrance stress and nurses’ work withdrawal behavior: a cross-sectional study

**DOI:** 10.3389/fpubh.2026.1806485

**Published:** 2026-05-08

**Authors:** Hui Ding, Yao Wang, Qin Zhang, Chenxia Song, Juan Chen, Guiqi Song

**Affiliations:** 1 Department of Child and Adolescent Psychology, The Affiliated Psychological Hospital of Anhui Medical University, Hefei, China; 2 Hefei Fourth People’s Hospital, Hefei, China; 3 School of Nursing, Anhui Medical University, Hefei, China; 4 Department of Nursing, The Affiliated Psychological Hospital of Anhui Medical University, Hefei, China; 5 Department of Education, The First Affiliated Hospital of University of Science and Technology of China (Anhui Provincial Hospital), Hefei, China

**Keywords:** challenge-hindrance stress, nurses, perceived organizational support, structural equation modeling, work withdrawal behavior

## Abstract

**Background:**

Nurses’ work withdrawal behavior has important implications for healthcare quality and patient safety. Perceived organizational support (POS) and challenge-hindrance stress have been identified as key factors associated with such behaviors. Although prior studies have examined the direct relationships between these factors and nurses’ work withdrawal behavior, the mediating role of perceived organizational support in the relationship between challenge-hindrance stress and work withdrawal behavior remains underexplored.

**Methods:**

Between June and July 2024, a cross-sectional survey was conducted among 3,420 registered nurses from 13 tertiary hospitals in Anhui Province, China. Participants completed validated self-report measures of challenge-hindrance stress, perceived organizational support, and work withdrawal behavior, along with a demographic questionnaire. Structural equation modeling (SEM) with bootstrapping (5,000 resamples) was used to examine the mediating role of perceived organizational support.

**Results:**

The mean scores for challenge-hindrance stress, perceived organizational support, and work withdrawal behavior were 32.76 ± 10.62, 46.40 ± 10.50, and 28.29 ± 9.92, respectively. SEM showed that perceived organizational support partially mediated the relationship between challenge-hindrance stress and work withdrawal behavior (*β* = 0.103, *p <* 0.001, 95% CI [0.087, 0.121]), accounting for 29.10% of the total effect (*β* = 0.354).

**Conclusion:**

These findings suggest that perceived organizational support may be an important organizational resource in the relationship between challenge-hindrance stress and nurses’ work withdrawal behavior. In high-pressure clinical settings, strengthening organizational support systems, such as recognition, resource provision, and career development opportunities, may be relevant to addressing nurses’ work withdrawal behavior and maintaining workforce stability and patient safety.

## 1 Introduction

Nurses are essential to achieving universal health coverage and the Sustainable Development Goals (SDGs). The China Nursing Development Plan (2021–2025) emphasizes that nursing is a cornerstone of the healthcare system, calling for strengthening of the nursing workforce to ensure high-quality nursing services (1). However, the shortage of nursing personnel remains a major global challenge. According to the World Health Organization, the global shortage of nurses is estimated at 5.9 million and may widen to 9 million by 2030 (2). In China, the total number of registered nurses reached 5.63 million by the end of 2023, with a nurse-to-population ratio of 4.0 per 1,000 people (3), which remains well below the OECD average of 9.0 per 1,000 (4). This shortage is particularly evident in Chinese tertiary hospitals, where nurses are often exposed to heavy workloads, high performance expectations, and complex clinical demands. Under such conditions, nurses may become psychologically and behaviorally disengaged from their work.

Work withdrawal behavior refers to psychological and behavioral disengagement from work and has become an important occupational issue among nurses in high-pressure clinical settings. It may reduce work engagement and job performance, undermine workforce stability, and threaten the quality of care and patient safety (5). Work withdrawal behavior generally includes two dimensions: psychological withdrawal and behavioral withdrawal. Psychological withdrawal is reflected in cognitive and emotional disengagement, such as reduced concentration, emotional indifference, lower work engagement, and increased turnover intention, whereas behavioral withdrawal is reflected in observable behaviors such as tardiness, absenteeism, early departure, and voluntary resignation (6). Previous studies have shown that withdrawal-related outcomes are common among nurses in high-intensity clinical environments. For example, turnover intention, which may reflect a broader tendency toward psychological disengagement from work, has been reported to be 27.7% among intensive care unit nurses and as high as 45% among emergency nurses worldwide (7, 8). In China, 37.3% of nurses have been reported to experience psychological withdrawal, and 13.7% exhibit moderate or higher levels of behavioral withdrawal (9). In addition, evidence suggests that work withdrawal behavior is influenced not only by external work stressors but also by internal psychological resources and cognitive appraisal (10).

Among the factors associated with work withdrawal behavior, job stress is considered one of the most important antecedents (11, 12). The Challenge-Hindrance Stress Model provides a useful framework for understanding how different stressors may shape occupational responses. Challenge stressors, such as high workload and expanded responsibilities, consume time and energy but may also involve opportunities for growth and achievement. In contrast, hindrance stressors, such as role conflict, bureaucratic procedures, insufficient resources, and unclear promotion pathways, are more likely to obstruct goal attainment and trigger negative outcomes (13). In Chinese tertiary hospitals, multiple demands related to clinical care, education, and research often coexist, and growth opportunities are frequently intertwined with obstructive demands (14, 15). Against this background, the present study modeled challenge-hindrance stress as a unified latent construct to represent the overall burden of work-related stress experienced by nurses, thereby more closely reflecting the reality of nursing work in tertiary hospitals.

If challenge-hindrance stress is associated with nurses’ work withdrawal behavior, it is important to understand further the mechanism through which this phenomenon occurs. Conservation of Resources (COR) theory posits that individuals are motivated to acquire, maintain, and protect valued resources, including energy, self-efficacy, emotional stability, and social support (16). According to this theory, resource loss has a stronger impact than resource gain. When resources are continuously depleted without timely replenishment, individuals are more likely to adopt defensive coping strategies to protect their remaining resources. In this sense, work withdrawal behavior can be understood as a negative occupational coping response that develops under sustained net resource loss (17). Although challenge stress may sometimes promote resource investment and potential gain, excessive or prolonged demands or insufficient coping resources may cause both challenge stress and hindrance stress to contribute to resource depletion and thus be associated with higher levels of work withdrawal behavior (13).

Perceived organizational support (POS) may be an important mechanism in this process. According to Social Exchange Theory (SET), employees adjust their attitudes and behaviors according to whether the organization values their contributions and cares about their well-being (18). POS reflects employees’ overall perception of the affective and instrumental support provided by the organization (19). For nurses, organizational support may not only buffer the adverse effects of stress but also provide a basis for resource recovery, renewed confidence, and re-engagement in work (20, 21). Conversely, when nurses are chronically exposed to high workload, conflict, and resource consumption, their resources may be continuously eroded, and they may perceive lower levels of organizational support as a stable and accessible external resource (22). Thus, POS may be more than a simple protective factor; it may function as a mediating organizational resource that shapes the relationship between challenge-hindrance stress and work withdrawal behavior.

In the present study, COR theory served as the overarching explanatory framework. Within this framework, the Challenge-Hindrance Stress Model was used to characterize job stressors, whereas Social Exchange Theory helped explain why POS may operate as an organizational resource linking stress to work withdrawal behavior. Overall, challenge-hindrance stress may accelerate resource depletion and be associated with higher levels of work withdrawal behavior, while higher POS may help mitigate resource loss and promote recovery and reinvestment, thereby being associated with lower levels of work withdrawal behavior. At present, studies explicitly conceptualizing POS as a mediating variable between challenge-hindrance stress and work withdrawal behavior remain limited, particularly among clinical nurses in Chinese tertiary hospitals, and empirical evidence for this mechanism is still insufficient.

Accordingly, this study aimed to examine the relationships among challenge-hindrance stress, perceived organizational support, and work withdrawal behavior among clinical nurses in Chinese tertiary hospitals. Specifically, the objectives were to: (1) describe the levels of challenge stress, hindrance stress, perceived organizational support, and work withdrawal behavior among clinical nurses in Chinese tertiary hospitals; (2) examine the relationship between overall challenge-hindrance stress, composed of challenge and hindrance stressors, and work withdrawal behavior; and (3) test the mediating role of perceived organizational support in the relationship between overall challenge-hindrance stress and work withdrawal behavior.

## 2 Methods

### 2.1 Study design

This study employed a cross-sectional survey design and was reported in accordance with the Strengthening the Reporting of Observational Studies in Epidemiology (STROBE) guidelines. The present study provides detailed descriptions of the study setting, participants, data collection procedures, variables, statistical methods, and ethical considerations.

### 2.2 Study setting and participants

This study was conducted between June and July 2024 using a cross-sectional survey design in 13 tertiary hospitals across 13 prefecture-level cities in Anhui Province, China. Anhui Province is considered to be at a mid-level of economic, social, and healthcare development in China. In the Chinese healthcare system, tertiary hospitals are large comprehensive medical institutions at the municipal, provincial, or national level, typically with more than 500 beds. They provide specialized healthcare services, play important roles in medical education and scientific research, and serve as regional medical centers.

A convenience sampling strategy was used. Eligible nurses were invited to participate through hospital-specific WeChat workgroups by trained research assistants from each participating hospital.

Participants were included if they met the following criteria: (1) were formally registered nurses currently engaged in clinical nursing practice; (2) had at least one year of professional experience; and (3) provided informed consent and voluntarily participated in the study. Exclusion criteria were (1) trainee nurses and visiting nurses from other institutions and (2) nurses who had been away from their clinical nursing posts for 3 months or longer at the time of the survey because of serious illness, maternity leave, study leave, or other similar reasons.

Sample size was estimated using G*Power 3.1.9 based on a fixed-model multiple linear regression. The significance level was set at *α* = 0.05, statistical power at 0.95, and the expected medium effect size at f^2^ = 0.15. In accordance with the initial study design, 15 predictor variables were included in the calculation, comprising 13 demographic variables, challenge-hindrance stress, and perceived organizational support. The minimum required sample size was estimated to be 199 participants. After accounting for a potential 20% attrition or invalid response rate during data collection, the final minimum required sample size was 239. A total of 3,420 valid questionnaires were included in the final analysis.

### 2.3 Measurement tools

Data were collected using a four-part questionnaire consisting of a general characteristics questionnaire, the Challenge-Hindrance Stress Scale, the Perceived Organizational Support Scale, and the Work Withdrawal Behavior Scale.

#### 2.3.1 General characteristics questionnaire

The general characteristics questionnaire was developed by the research team and included 13 items, such as gender, age, marital status, fertility status, years of work experience, educational level, professional title, employment type, monthly night shift frequency, monthly performance-based income, work department, current position, and hospital position promotion system.

#### 2.3.2 Work Withdrawal Behavior Scale

Work withdrawal behavior was measured using the Work Withdrawal Behavior Scale developed by Lehman (23) and adapted into Chinese by Yang Yazhong (24). The scale consists of two dimensions: psychological withdrawal (8 items) and behavioral withdrawal (4 items). Each item is rated on a 5-point Likert scale ranging from 1 (never) to 5 (always). The total score ranges from 12 to 60, with higher scores indicating more frequent work withdrawal behaviors among nurses. In the present study, the Cronbach’s *α* coefficient for this scale was 0.887.

#### 2.3.3 Challenge-hindrance stress scale

Challenge-hindrance stress was measured using the scale developed by Cavanaugh et al. (13) and translated and revised into Chinese by Zhang Yunli (25). The scale consists of two dimensions: challenge stressors (6 items) and hindrance stressors (5 items). Each item is rated on a 5-point Likert scale ranging from 1 (strongly disagree) to 5 (strongly agree). The total score ranges from 11 to 55, with higher scores indicating higher levels of perceived stress among nurses. In the present study, the Cronbach’s *α* coefficient for this scale was 0.874.

#### 2.3.4 Perceived organizational support scale

Perceived organizational support was assessed using the scale revised by Zuo Hongmei (26). The scale consists of two dimensions: affective support (10 items) and instrumental support (3 items). Each item is rated on a 5-point Likert scale ranging from 1 (strongly disagree) to 5 (strongly agree). The total score ranges from 13 to 65, with higher scores indicating higher levels of perceived organizational support. In the present study, the Cronbach’s *α* coefficient for this scale was 0.968.

### 2.4 Data collection procedure

Between June and July 2024, an online questionnaire was developed using the Wenjuanxing platform, and a unique QR code was generated for distribution. Prior to data collection, the principal investigator provided standardized online training for 13 research assistants, one from each participating hospital, covering the study objectives, ethical considerations, data collection procedures, and quality control requirements.

Following the training, each research assistant distributed the survey QR code to eligible nurses through hospital-specific WeChat workgroups. The introduction to the survey clearly described the study purpose, inclusion and exclusion criteria, the voluntary nature of participation, the informed consent process, and the contact information of the research team. Participants who did not meet the eligibility criteria were automatically screened out through the embedded survey logic and were unable to proceed to the main questionnaire.

All questionnaire items were set as mandatory, and the platform allowed only one submission per IP address to prevent duplicate entries. The survey remained open for 10 days. During this period, response times and response patterns were reviewed to identify careless or invalid responses, and questionnaires deemed invalid were excluded from the final analysis. A total of 4,083 responses were initially collected, of which 3,420 valid questionnaires were retained for analysis. Because the survey link was distributed through hospital-specific WeChat workgroups, the exact number of eligible nurses who received and viewed the invitation could not be determined; therefore, a precise response rate could not be calculated. For the same reason, detailed reasons for non-participation were not available.

### 2.5 Data analysis

Data were analyzed using SPSS 26.0 and AMOS 24.0. Frequencies, percentages, means, and standard deviations were used to describe participants’ general characteristics and the levels of the main study variables. Harman’s single-factor test was conducted to assess common method bias. Independent-samples t tests or one-way analysis of variance was used to compare differences in work withdrawal behavior scores across demographic characteristics, and hierarchical regression analysis was performed to examine the predictive effects of challenge-hindrance stress and perceived organizational support on nurses’ work withdrawal behavior. Pearson’s correlation analysis was used to examine associations among the main study variables. Confirmatory factor analysis (CFA) was conducted to evaluate the fit of the measurement model, and structural equation modeling (SEM) was used to test the mediating role of perceived organizational support in the relationship between challenge-hindrance stress and work withdrawal behavior. The indirect effect was examined using bootstrapping with 5,000 resamples, which is widely recommended for mediation analysis. In this study, challenge stress and hindrance stress were modeled as indicators of the overall burden of work-related stress. Model fit was evaluated using the chi-square/df ratio (CMIN/DF), RMSEA, CFI, NFI, TLI, and SRMR. A two-tailed *p <* 0.05 was considered statistically significant.

### 2.6 Ethical considerations

This study was conducted in accordance with the ethical principles of the Declaration of Helsinki and was approved by the Ethics Committee of the Affiliated Mental Health Hospital of Anhui Medical University (Approval No. HFSY-IRB-YJ-KYXM-DH (2024–054-00)). Before completing the questionnaire, all participants read the informed consent form and were fully informed of the study purpose, content, and the voluntary nature of participation. Only those who provided informed consent were allowed to proceed to the formal survey. Participant anonymity and data confidentiality were ensured throughout the study. All data were used solely for research purposes and did not involve disclosure of personal privacy. Participants were free to withdraw from the study at any time without penalty.

## 3 Results

### 3.1 Common method bias test

Harman’s single-factor test was conducted to preliminarily assess the potential influence of common method bias. The results showed that, in the unrotated exploratory factor analysis, the first factor accounted for 36.45% of the total variance, which was below the commonly used threshold of 40% (27), suggesting that serious common method bias was unlikely in the present study.

### 3.2 General characteristics of the participants and univariate analysis

A total of 3,420 nurses were included in the study. As shown in Table 1, most participants were female (96.32%), aged 30–39 years (51.61%), married (79.53%), and had a bachelor’s degree (84.21%). The majority were contract staff (68.60%) and were primary nurses (72.57%). Nearly half of the participants (48.01%) reported a monthly performance-based income below CNY 5,000, and 55.00% worked 5–7 night shifts per month. In addition, 89.77% reported that their hospitals had a formal position promotion system. Univariate analysis showed that work withdrawal behavior differed significantly by gender, years of work experience, educational level, professional title, employment type, monthly night shift frequency, monthly performance-based income, work department, current position, and hospital position promotion system (all *p <* 0.05).

**Table 1:** General characteristics of the participants and univariate analysis of work withdrawal behavior (*n* = 3,420).

Variable	Group	*n* (%)	Mean ± SD	t/F	*p*
Gender	Female	3,294 (96.32)	28.13 ± 9.84	−4.533	<0.001
	Male	126 (3.68)	32.62 ± 10.96		
Age (years)	<30	863 (25.23)	27.91 ± 9.75	1.668	0.172
	30–39	1,765 (51.61)	28.66 ± 10.06		
	40–50	613 (17.92)	27.96 ± 9.71		
	>50	179 (5.23)	27.69 ± 9.90		
Marital status	Unmarried	636 (18.60)	28.61 ± 10.03	1.228	0.298
	Married	2,720 (79.53)	28.17 ± 9.83		
	Divorced	59 (1.73)	30.34 ± 11.90		
	Widowed	5 (0.15)	29.60 ± 14.71		
Fertility status	Without children	891 (26.05)	28.19 ± 9.89	−0.358	0.721
	With children	2,529 (73.95)	28.33 ± 9.93		
Years of work experience	1–5	607 (17.75)	27.83 ± 9.88	2.505	0.040
	6–10	927 (27.11)	28.96 ± 10.30		
	11–15	933 (27.28)	28.57 ± 9.87		
	16–20	409 (11.96)	27.65 ± 9.44		
	>20	544 (15.91)	27.68 ± 9.65		
Educational level	College or below	517 (15.12)	27.23 ± 9.77	18.533	<0.001
	Bachelor’s degree	2,880 (84.21)	28.39 ± 9.89		
	Master’s degree or above	23 (0.67)	39.70 ± 8.33		
Professional title	Nurse	302 (8.83)	27.31 ± 9.94	5.799	0.001
	Senior nurse	1,137 (33.25)	27.57 ± 9.79		
	Nurse-in-charge	1,770 (51.75)	28.75 ± 9.88		
	Associate chief nurse or above	211 (6.17)	29.75 ± 10.55		
Employment type	Permanent staff	689 (20.15)	28.68 ± 10.17	9.813	<0.001
	Contract staff	2,346 (68.60)	27.82 ± 9.79		
	Personnel agency staff	338 (9.88)	30.86 ± 9.85		
	Labor dispatch staff	47 (1.37)	27.55 ± 9.92		
Monthly night shift frequency	5–7 shifts/month	1,881 (55.00)	28.51 ± 10.01	3.748	0.011
	8–10 shifts/month	530 (15.50)	29.09 ± 10.33		
	11–20 shifts/month	249 (7.28)	27.71 ± 9.40		
	>20 shifts/month	760 (22.22)	27.40 ± 9.49		
Monthly performance-based income (CNY)	<5,000	1,642 (48.01)	27.21 ± 9.56	20.475	<0.001
	5,000–10,000	1,556 (45.50)	29.13 ± 10.17		
	>10,000	222 (6.49)	30.38 ± 9.82		
Work department	Emergency department	155 (4.53)	29.47 ± 10.35	3.671	0.001
	Intensive care unit	144 (4.21)	29.81 ± 9.93		
	Internal medicine department	1,032 (30.18)	28.75 ± 10.10		
	Surgery department	752 (21.99)	28.67 ± 9.93		
	Pediatrics department	234 (6.84)	27.83 ± 9.77		
	Obstetrics and gynecology department	205 (5.99)	27.25 ± 9.96		
	Operating room	154 (4.50)	29.16 ± 10.28		
	Other department*^&^*	744 (21.75)	26.99 ± 9.37		
Current position	Primary nurse	2,482 (72.57)	28.34 ± 10.03	3.432	0.016
	Team leader/clinical instructor	358 (10.47)	28.56 ± 9.68		
	Ward head nurse/deputy head nurse	325 (9.50)	29.02 ± 9.73		
	Others*	255 (7.46)	26.51 ± 9.20		
Hospital position promotion system	Yes	3,070 (89.77)	28.05 ± 9.84	9.205	*<*0.001
	No	88 (2.57)	31.11 ± 10.45		
	Not sure	262 (7.66)	30.17 ± 10.25		

& includes the intravenous admixture center, central sterile supply department, and similar units; * includes infection control nurses, treatment nurses, and similar positions.

### 3.3 Scores of challenge-hindrance stress, perceived organizational support, and work withdrawal behavior

The mean total scores of challenge-hindrance stress, perceived organizational support, and work withdrawal behavior among nurses were 32.76 ± 10.62, 46.40 ± 10.50, and 28.29 ± 9.92, respectively. Detailed scores for each scale and its subscales are presented in Table 2.

**Table 2:** Scores of challenge-hindrance stress, perceived organizational support, and work withdrawal behavior among nurses.

Variables	Items	Score *(M ± SD)*	Score of items *(M ± SD)*
Challenge-hindrance stress	11	32.76 ± 10.62	2.98 ± 0.97
Challenge stress	6	18.29 ± 5.78	3.05 ± 0.96
Hindrance stress	5	14.46 ± 5.33	2.89 ± 1.07
Perceived organizational support	13	46.40 ± 10.50	3.57 ± 0.81
Emotional support	10	35.05 ± 8.45	3.51 ± 0.85
Instrumental support	3	11.35 ± 2.40	3.78 ± 0.80
Work withdrawal behavior	12	28.29 ± 9.92	2.36 ± 0.83
Psychological withdrawal	8	19.63 ± 6.93	2.45 ± 0.87
Behavioral withdrawal	4	8.66 ± 3.54	2.17 ± 0.89

### 3.4 Correlations between challenge-hindrance stress, perceived organizational support, and work withdrawal behavior

Pearson’s correlation analysis showed that challenge-hindrance stress was negatively correlated with perceived organizational support (*r* = −0.312, *p <* 0.001) and positively correlated with work withdrawal behavior (*r* = 0.308, *p <* 0.001). Perceived organizational support was negatively correlated with work withdrawal behavior (*r* = −0.345, *p <* 0.001) (Table 3).

**Table 3:** Pearson correlation matrix among challenge-hindrance stress, perceived organizational support, and work withdrawal behavior.

Variable	1	2	3
1. Challenge-hindrance stress	1		
2. Perceived organizational support	−0.312^***^	1	
3. Work withdrawal behavior	0.308^***^	−0.345^***^	1

***p < 0.001.

### 3.5 Hierarchical regression analysis of work withdrawal behavior

Before hierarchical regression analysis, multicollinearity was assessed using variance inflation factor (VIF) and tolerance values. All VIF values were below 3.0 (range: 1.011–2.536), and all tolerance values exceeded 0.390, indicating that there was no serious multicollinearity among the predictors (28).

Hierarchical regression analysis was performed with work withdrawal behavior score as the dependent variable. In Step 1, the 10 demographic and work-related variables that were statistically significant in the univariate analyses were entered as control variables. Among these, years of work experience, educational level, professional title, monthly night shift frequency, and monthly performance-based income were entered as ordinal variables. Gender, employment type, current position, work department, and hospital position promotion system were treated as nominal variables and dummy coded, with female nurses, permanent staff, primary nurses, other departments, and hospitals with a position promotion system as the reference categories. The results of Step 1 showed that gender, years of work experience, professional title, monthly performance-based income, contract employment, the absence of a hospital position promotion system, uncertainty about the hospital position promotion system, and working in the internal medicine department were significant predictors of work withdrawal behavior (all *p <* 0.05).

In Step 2, challenge-hindrance stress was entered into the model and was positively associated with work withdrawal behavior (*B* = 0.272, *β* = 0.291, *p <* 0.001). The explained variance increased to 12.7% (ΔR^2^ = 0.083, *p <* 0.001), and the adjusted R^2^ increased to 0.121. In Step 3, perceived organizational support was further added and was negatively associated with work withdrawal behavior (*B* = −0.268, *β* = −0.284, *p <* 0.001). The explained variance increased significantly to 19.3% (ΔR^2^ = 0.067, *p <* 0.001), and the adjusted R^2^ increased to 0.188. These findings suggest that perceived organizational support explained additional variance in work withdrawal behavior and attenuated the association between challenge-hindrance stress and work withdrawal behavior, which may indicate a potential mediating role of perceived organizational support (Table 4).

**Table 4:** Hierarchical multiple linear regression analysis of factors associated with work withdrawal behavior among clinical nurses.

Variable	Model 1				Model 2				Model 3			
	*B*	*β*	t	*p*	*B*	*β*	t	*p*	*B*	*β*	t	*p*
Constant	19.779		11.989	*<* 0.001	11.731		7.156	*<* 0.001	27.627		15.014	*<* 0.001
Gender	4.195	0.080	4.482	*<* 0.001	3.751	0.071	4.191	*<* 0.001	3.084	0.059	3.580	*<* 0.001
Years of work experience	−0.676	−0.089	−3.331	0.001	−0.714	−0.094	−3.681	*<* 0.001	−0.721	−0.095	−3.864	*<* 0.001
Educational level	0.381	0.014	0.768	0.443	0.218	0.008	0.459	0.646	0.149	0.006	0.327	0.743
Professional title	1.511	0.113	4.374	*<* 0.001	1.326	0.099	4.011	*<* 0.001	1.142	0.085	3.593	*<* 0.001
Monthly night shift frequency	−0.268	−0.033	−1.529	0.126	−0.104	−0.013	−0.620	0.535	−0.043	−0.005	−0.267	0.790
Monthly performance-based income	1.221	0.075	4.170	*<* 0.001	1.228	0.076	4.387	*<* 0.001	1.466	0.090	5.441	*<* 0.001
Employment type												
Contract staff	−1.226	−0.057	−2.586	0.010	−0.825	−0.039	−1.817	0.069	−0.841	−0.039	−1.927	0.054
Personnel agency staff	1.011	0.030	1.482	0.138	0.964	0.029	1.479	0.139	0.773	0.023	1.234	0.217
Labor dispatch staff	−0.060	−0.001	−0.039	0.969	0.169	0.002	0.115	0.909	−0.570	−0.007	−0.402	0.688
Current position												
Team leader/clinical instructor	−0.323	−0.010	−0.566	0.571	−0.608	−0.019	−1.115	0.265	−0.186	−0.006	−0.354	0.723
Ward head nurse/deputy head nurse	0.270	0.008	0.381	0.703	−0.295	−0.009	−0.436	0.663	1.168	0.035	1.776	0.076
Others	−0.798	−0.021	−1.160	0.246	−0.523	−0.014	−0.795	0.427	−0.183	−0.005	−0.290	0.772
Hospital position promotion system												
No	2.527	0.040	2.394	0.017	1.613	0.026	1.596	0.111	−0.787	−0.013	−0.801	0.423
Not sure	2.169	0.058	3.444	0.001	1.514	0.041	2.510	0.012	−0.037	−0.001	−0.063	0.950
Work department												
Emergency department	1.227	0.026	1.368	0.171	1.059	0.022	1.235	0.217	0.703	0.015	0.853	0.394
Intensive care unit	0.870	0.018	0.935	0.350	1.173	0.024	1.318	0.187	1.150	0.023	1.344	0.179
Internal medicine department	1.101	0.051	2.220	0.026	0.978	0.045	2.063	0.039	0.941	0.044	2.064	0.039
Surgery department	0.940	0.039	1.763	0.078	1.078	0.045	2.114	0.035	0.955	0.040	1.948	0.051
Pediatrics department	0.452	0.012	0.602	0.547	0.371	0.009	0.517	0.605	0.418	0.011	0.606	0.545
Obstetrics and gynecology department	−0.583	−0.014	−0.740	0.459	−0.346	−0.008	−0.459	0.646	−0.216	−0.005	−0.298	0.766
Operating room	0.942	0.020	1.074	0.283	0.993	0.021	1.185	0.236	1.195	0.025	1.484	0.138
Challenge-hindrance stress					0.272	0.291	17.949	<0.001	0.192	0.206	12.525	*<* 0.001
Perceived organizational support									−0.268	−0.284	−16.734	*<* 0.001
Model fit	Model 1				Model 2				Model 3			
*F*	7.462				22.439				35.401			
*p*	*<* 0. 001				*<* 0. 001				*<* 0. 001			
R^2^	0.044				0.127				0.193			
Adjusted R^2^	0.038				0.121				0.188			

### 3.6 Structural equation modeling of the mediating effect

Structural equation modeling (SEM) was conducted to examine the mediating effect of perceived organizational support on the relationship between challenge-hindrance stress and work withdrawal behavior. The model included three latent variables—challenge-hindrance stress, perceived organizational support, and work withdrawal behavior—and six observed indicators: challenge stress, hindrance stress, affective support, instrumental support, psychological withdrawal, and behavioral withdrawal.

First, confirmatory factor analysis (CFA) was performed to evaluate the measurement model. The fit indices indicated a good model fit: CMIN/DF = 2.505, RMSEA = 0.013, CFI = 0.999, NFI = 0.996, TLI = 0.992, SRMR = 0.012. The standardized factor loadings of the observed indicators on their corresponding latent variables ranged from 0.856 to 0.967, and all were statistically significant (*p <* 0.001), supporting the adequacy of the measurement model.

**Figure 1:**
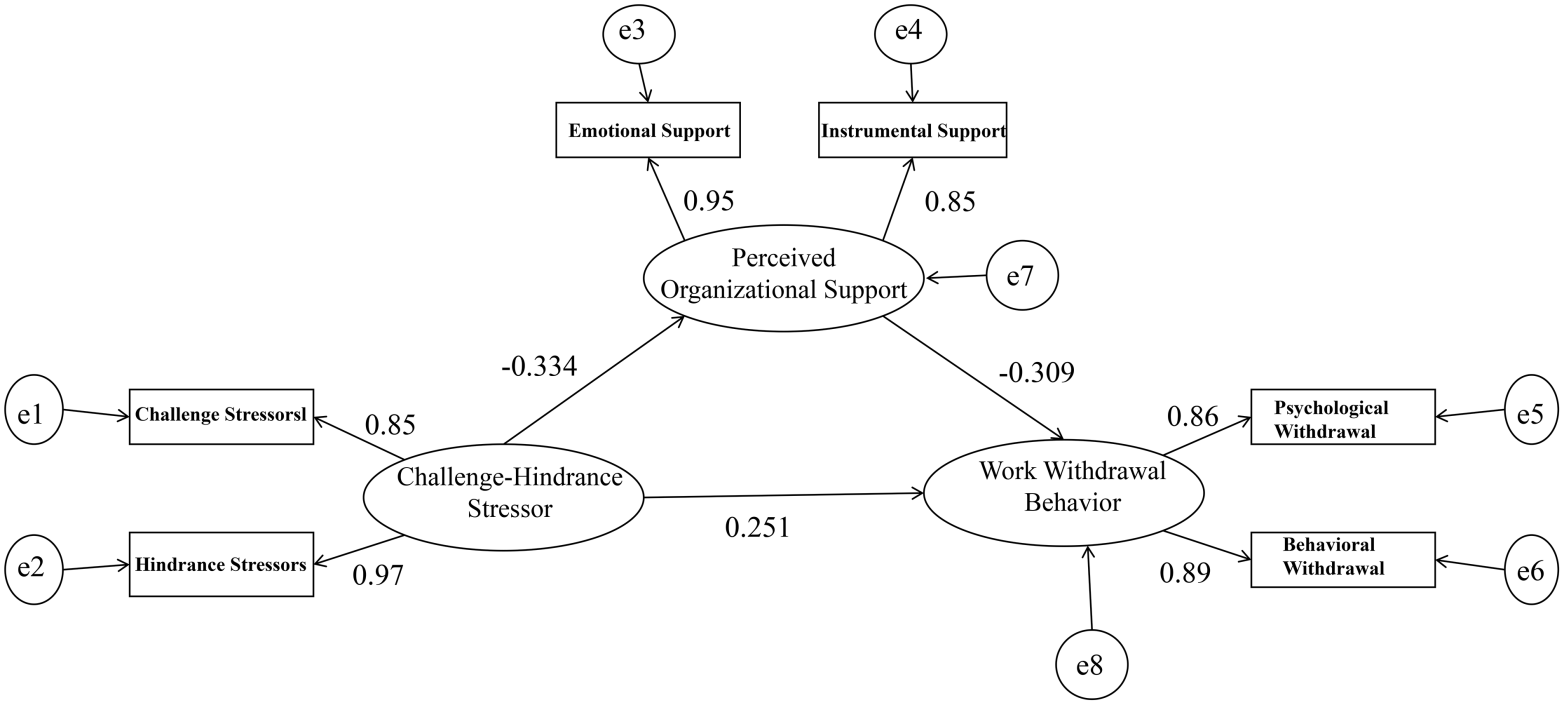
Structural equation model depicting the mediating role of perceived organizational support in the relationship between challenge-hindrance stress and work withdrawal behavior among nurses.

The structural model showed that challenge-hindrance stress had a significant direct positive effect on work withdrawal behavior (*β* = 0.251, *p <* 0.001). In addition, challenge-hindrance stress significantly and negatively predicted perceived organizational support (*β* = −0.334, *p <* 0.001), whereas perceived organizational support significantly and negatively predicted work withdrawal behavior (*β* = −0.309, *p <* 0.001). Bootstrapping with 5,000 resamples further showed that the indirect effect of challenge-hindrance stress on work withdrawal behavior through perceived organizational support was statistically significant (*β* = 0.103, 95% CI [0.087, 0.121], *p <* 0.001). The standardized path coefficients from challenge-hindrance stress to perceived organizational support and from perceived organizational support to work withdrawal behavior suggested associations of modest magnitude. Although the standardized indirect effect was smaller in size, it accounted for 29.1% of the total effect (total effect: β = 0.354), further supporting the partial mediating role of perceived organizational support (Figure 1; Table 5).

**Table 5:** Direct, indirect, and total standardized effects of challenge-hindrance stress on work withdrawal behavior in the structural equation model.

Path	*β*	SE	95% CI (Lower)	95% CI (Upper)
Direct effect: Challenge-hindrance stress → Work withdrawal behavior	0.251	0.020	0.212	0.292
Indirect effect: Challenge-hindrance stress → Perceived organizational support → Work withdrawal behavior	0.103	0.016	0.087	0.121
Total effect: Challenge-hindrance stress → Work withdrawal behavior	0.354	0.018	0.319	0.391

## 4 Discussion

This study found that the total work withdrawal behavior score among nurses in tertiary hospitals in Anhui Province was 28.29 ± 9.92. Considering the scale range of 12–60 and its theoretical midpoint of 36, the overall level of work withdrawal behavior in this sample can be regarded as slightly below moderate. Compared with previous domestic studies, the present finding was lower than that reported by Hu Shenmian among nurses in tertiary hospitals, but higher than that reported by Wang Yan among clinical nurses in public hospitals in Ningxia, suggesting that the level of work withdrawal behavior may vary across regions and sample sources (29, 30). Such differences may be partly related to regional variations in nursing workforce allocation and the functional positioning of medical institutions. A previous study based on panel data from 31 provinces in China showed that the nursing workforce allocation in Anhui Province was below the national average, which may indicate relatively constrained nursing human resources in this region and potentially heavier workloads for nurses (31). In addition, tertiary hospitals typically undertake multiple responsibilities, including the treatment of critically ill patients, the management of complex cases, and teaching and research tasks, all of which may place greater demands on nurses’ professional competence and psychological resilience. These contextual factors may help explain the level of work withdrawal behavior observed in the present study.

This study also found that gender, years of work experience, professional title, monthly performance-based income, and working in the internal medicine department were significantly associated with nurses’ work withdrawal behavior in the final model (all *p* < 0.05), suggesting that work withdrawal behavior may vary across individual and job-related characteristics. Specifically, the association with years of work experience was broadly consistent with previous findings, whereas the patterns observed for professional title and monthly performance-based income differed somewhat from traditional expectations (32, 33). From the perspective of COR theory, nurses with longer work experience tend to accumulate richer occupational resources, including clinical experience, self-efficacy, and problem-solving abilities. When facing work pressure, these nurses are more likely to maintain a relatively stable occupational state and therefore exhibit lower levels of work withdrawal behavior (34). By contrast, nurses with higher professional titles and higher monthly performance-based income often assume greater responsibilities related to clinical management, teaching, research, and team coordination. The increased job demands and role responsibilities they face may place them in a state of sustained resource depletion, thereby increasing the risk of work withdrawal behavior. Gender differences in work withdrawal behavior may reflect variations in workplace role expectations, stress perception, and coping styles across groups (35). In addition, nurses working in the internal medicine department may be more vulnerable to work withdrawal behavior because of heavy workloads, complex patient care tasks, and prolonged physical and emotional demands (36). These findings indicate that when identifying groups at high risk of work withdrawal behavior, nurse managers should take into account nurses’ individual characteristics, career development stages, and department-specific workload characteristics and develop more targeted support strategies accordingly. In practice, this may include closer monitoring of nurses in high-demand departments, providing tailored support for nurses at different career stages, and adjusting workload and role expectations for nurses with heavier managerial, teaching, or coordination responsibilities.

This study further found that challenge-hindrance stress was positively associated with nurses’ work withdrawal behavior, and the SEM analysis showed a significant positive direct effect (*β* = 0.251, 95% CI [0.212, 0.292]). This finding is broadly consistent with previous studies and suggests that a greater overall burden of work-related stress may be associated with higher levels of work withdrawal behavior among nurses (37). In Chinese tertiary hospitals, nurses often undertake multiple responsibilities related to clinical care, teaching, and research, making their stress experience inherently complex. Such demands include both challenge stressors, which may under certain conditions facilitate growth and professional development, and hindrance stressors arising from institutional procedures, role conflict, and managerial barriers. From the perspective of the Challenge-Hindrance Stress Model and COR theory, although moderate challenge stress may be associated with positive occupational outcomes in some circumstances, once job demands accumulate beyond individuals’ available resources and coping capacity, both challenge and hindrance stressors may contribute to sustained resource depletion and be associated with higher levels of work withdrawal behavior (13, 16). These findings suggest that nursing managers should adopt a more integrated approach to stress management, taking into account the combined effects of multiple stressors rather than focusing on only one type of stress. Practical measures may include optimizing task allocation, reducing unnecessary administrative burden, improving scheduling flexibility, and providing timely support for nurses working in persistently high-pressure environments.

The results further showed that challenge-hindrance stress was negatively associated with perceived organizational support, and the SEM path coefficient showed a negative relationship between the two variables (*β* = −0.334). This finding is broadly consistent with previous research, suggesting that higher levels of work-related stress may weaken nurses’ perceptions of organizational support (38). According to COR theory, when individuals remain in a state of high resource expenditure over time, their available resources decrease, and their sensitivity to and ability to mobilize external resources may also decline. At the same time, social exchange theory suggests that employees form overall evaluations of their organization based on the resources and support it provides (18). When nurses are exposed to sustained high workloads without receiving adequate emotional care, institutional support, and resource provision, their perceived organizational support may decline. From a management perspective, efforts to address nurses’ occupational stress should not be limited to workload and workflow adjustments alone but should also attend to nurses’ subjective perceptions of organizational support by improving management transparency, optimizing resource provision, strengthening emotional support, and ensuring that nurses can access fair promotion opportunities, adequate staffing, and timely communication from supervisors.

Perceived organizational support was also negatively associated with nurses’ work withdrawal behavior, and the SEM path coefficient likewise indicated a negative relationship (*β* = −0.309). This finding is broadly consistent with previous research and suggests that higher perceived organizational support may be associated with lower levels of work withdrawal behavior among nurses (39). According to COR theory, organizational support can be regarded as an important external resource for nurses, and its association with lower levels of work withdrawal behavior may be explained by its role in replenishing the emotional and cognitive resources consumed under high work demands (16). From the perspective of social exchange theory, when nurses perceive that their organization values their contributions and cares about their well-being, they may be more likely to respond with greater engagement rather than withdrawal (18). Thus, organizational support is not merely a background feature of the work environment but may also represent an important resource condition shaping nurses’ occupational behavior. From a nursing management perspective, enhancing perceived organizational support may be an important entry point for addressing work withdrawal behavior among nurses. Hospitals may strengthen nurses’ sense of organizational belonging and professional identity by improving career development pathways, establishing fair and transparent promotion systems, strengthening humanistic care, and providing practical resource support. In particular, visible recognition from supervisors, accessible psychological support, and consistent organizational communication may be helpful in reducing withdrawal tendencies among nurses.

More importantly, perceived organizational support partially mediated the relationship between challenge-hindrance stress and nurses’ work withdrawal behavior, as the indirect effect was significant (*β* = 0.103, 95% CI [0.087, 0.121]) while the direct effect remained significant. This finding suggests that challenge-hindrance stress may be associated with work withdrawal behavior not only directly, but also indirectly through nurses’ perceptions of organizational support. It extends previous studies that have examined the independent roles of stress or organizational support and further indicates that perceived organizational support may represent an important mechanism linking stress and work withdrawal behavior. From the perspectives of COR theory and social exchange theory (16, 18), high levels of stress may be associated with increased resource depletion and reduced perceptions of support, whereas organizational support, as a key external resource, may contribute to resource replenishment, recovery, and reinvestment. Perceived organizational support may therefore play an important role in the process linking stress to work withdrawal behavior. For nursing management practice, these findings suggest that reducing job stress alone or focusing solely on individual adaptation may be insufficient to effectively address nurses’ work withdrawal behavior. Managers may also need to strengthen organizational support systems and provide more stable emotional support and resource provision to buffer the adverse effects of stress and reduce the likelihood of work withdrawal behavior. More specifically, hospitals may consider establishing structured support programs for nurses in high-stress departments, improving access to supervisory feedback and organizational recognition, and integrating stress management with resource-support interventions rather than addressing these issues separately.

It should also be noted that challenge stressors and hindrance stressors were deliberately modeled as a unified latent construct in the present study, based on the assumption that nurses in high-pressure clinical settings often experience these demands concurrently. Although this approach is useful for capturing the overall stress burden and its associations with perceived organizational support and work withdrawal behavior, it does not allow the potentially divergent effects of challenge and hindrance stressors to be distinguished. Future research should further compare one-factor and two-factor models to examine whether challenge and hindrance stressors exert distinct relationships with perceived organizational support and work withdrawal behavior.

## 5 Conclusion

In conclusion, nurses in tertiary hospitals in Anhui Province showed a slightly below-moderate level of work withdrawal behavior. Challenge-hindrance stress was positively associated with work withdrawal behavior, whereas perceived organizational support was negatively associated with work withdrawal behavior. In addition, perceived organizational support partially mediated the relationship between challenge-hindrance stress and work withdrawal behavior. These findings suggest that work withdrawal behavior among nurses should be understood not only in relation to overall stress burden but also in relation to the availability of organizational support resources. From a practical perspective, nursing managers may need to pay closer attention to the combined impact of multiple work stressors and strengthen organizational support systems to better address work withdrawal behavior and maintain workforce stability and quality of care.

### 5.1 Strengths and limitations

This study has several strengths. First, it was based on a large multicenter sample of 3,420 nurses from 13 tertiary hospitals across 13 prefecture-level cities in Anhui Province, which improves the representativeness of the findings within the provincial tertiary-care context. Second, by combining hierarchical regression analysis and structural equation modeling, the study was able to examine both demographic and work-related correlates and the hypothesized mediating mechanism in a complementary manner. Third, the study was guided by an integrated theoretical framework based on Conservation of Resources theory, the Challenge-Hindrance Stress Model, and Social Exchange Theory, which strengthened the interpretation of the observed relationships and their practical implications for nursing management.

This study has several limitations. First, all variables were measured using self-report questionnaires at a single time point. Although Harman’s single-factor test suggested that serious common method bias was unlikely, reporting bias and residual common method variance cannot be completely ruled out. Future studies could incorporate multisource or more objective assessments of work withdrawal behavior, such as supervisor ratings, attendance records, and peer or patient evaluations. Second, the sample was drawn from tertiary hospitals in Anhui Province, which may limit the generalizability of the findings to other hospital levels, regions, or healthcare settings. Third, this study deliberately modeled challenge stressors and hindrance stressors as a unified latent construct, based on the consideration that nurses in high-pressure clinical settings often experience both types of demands concurrently. Although this composite approach is useful for capturing overall stress burden and its associations with perceived organizational support and work withdrawal behavior, it does not allow the potentially divergent effects of challenge and hindrance stressors to be distinguished. Future research should further compare one-factor and two-factor models or apply multi-group structural equation modeling to examine these relationships more precisely. Finally, the cross-sectional design limits causal inference. Future longitudinal, repeated-measures, or mixed-methods studies are needed to further clarify the temporal ordering and underlying mechanisms linking challenge-hindrance stress, perceived organizational support, and work withdrawal behavior.

## Data Availability

The original contributions presented in the study are included in the article/supplementary material, further inquiries can be directed to the corresponding author.
